# Inhibition of ANXA2 activity attenuates epileptic susceptibility and GluA1 phosphorylation

**DOI:** 10.1111/cns.14295

**Published:** 2023-06-11

**Authors:** Limin Ma, Qingyuan Wu, Jinxian Yuan, You Wang, Peng Zhang, Qiankun Liu, Dandan Tan, Minxue Liang, Yangmei Chen

**Affiliations:** ^1^ Department of Neurology The Second Affiliated Hospital of Chongqing Medical University Chongqing China; ^2^ Department of Neurology Chongqing University Three Gorges Hospital Chongqing China

**Keywords:** AMPAR, ANXA2, epilepsy, excitatory synaptic transmission, GluA1, seizure

## Abstract

**Introduction:**

Annexin A2 (ANXA2) participates in the pathology of a variety of diseases. Nevertheless, the impact of ANXA2 on epilepsy remains to be clarified.

**Aims:**

Hence, the study aimed at investigating the underlying role of ANXA2 in epilepsy through behavioral, electrophysiological, and pathological analyses.

**Results:**

It was found that ANXA2 was markedly upregulated in the cortical tissues of temporal lobe epilepsy patients (TLE), kainic acid (KA)‐induced epilepsy mice, and in a seizure‐like model in vitro. ANXA2 silencing in mice suppressed first seizure latency, number of seizures, and seizure duration in behavioral analysis. In addition, abnormal brain discharges were less frequent and shorter in the hippocampal local field potential (LFP) record. Furthermore, the results showed that the frequency of miniature excitatory postsynaptic currents was decreased in ANXA2 knockdown mice, indicating that the excitatory synaptic transmission is reduced. Co‐immunoprecipitation (COIP) experiments demonstrated that ANXA2 interacted with the α‐amino‐3‐hydroxy‐5‐methyl‐4‐isoxazolepropionic acid receptor (AMPAR) subunit GluA1. Moreover, ANXA2 knockdown decreased GluA1 expression on the cell surface and its phosphorylation onserine 831 and serine 845, related to the decreased phosphorylation levels mediated by protein kinases A and C (PKA and PKC).

**Conclusions:**

This study covers a previously unknown and key function of ANXA2 in epilepsy. These findings indicate that ANXA2 can regulate excitatory synaptic activity mediated by AMPAR subunit GluA1 to improve seizure activity, which can provide novel insights for the treatment and prevention of epilepsy.

## Graphical abstract

ANXA2 promotes phosphorylation of the AMPAR subunits GluR1‐S845 and GluR1‐S831, resulting in increased AMPAR membrane protein expression and enhanced excitatory synaptic transmission in neurons, which is involved in epilepsy formation.

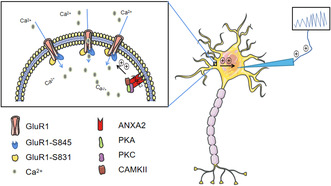

## 1 BACKGROUND

Epilepsy is a common disorder of the nervous system, associated with a highly synchronized firing of abnormal brain neurons. There are currently about 65 million people with epilepsy worldwide, and about 2.4 million new cases are recorded each year.^1^, ^2^ Pharmacotherapy is currently the mainstay of treatment for epilepsy, and there are still 20%–30% of patients who develop intractable epilepsy. Currently, the oncogenesis of epilepsy is not entirely known, and further investigation is required.

Anomalous excitatory synaptic transmission is involved in the pathogenesis of epileptic episodes.^3^, ^4^ The AMPAR is distributed widely throughout the brain and is a key glutamate receptor responsible for synaptic plasticity and excitatory synaptic transmission in epilepsy. GluA1 is one of the important AMPAR subunits, in which its number and distribution are involved in the excitatory activity of neurons.^5^, ^6^ Phosphorylation sites on its C‐terminal are the primary regulators of GluA1 distribution on the cell membrane. There are four reported phosphorylation sites related to GluA1 function, and the most prominent are S831 and S845, associated with AMPAR‐mediated excitatory synaptic activity.^7^, ^8^ The phosphorylation of S831 and S845 mediated by AMPAR may modulate synaptic transmission strength in neuronal networks.^9^, ^10^ S831 is phosphorylated by calcium‐/calmodulin‐dependent protein kinase II (CaMKII) and PKC, whereas S845 is phosphorylated by PKA. Meanwhile, GluA1 dephosphorylation is mediated by protein phosphatase 1, 2A, and 2B (PP1, PP2A, and PP2B).^11^, ^12^ Elevated levels of GluA1 phosphorylation on S831 and S845 have been proven to be significantly associated with seizure rate and cognitive impairment in neonatal epilepsy patients.^13^, ^14^ They provided evidence for the phosphorylation of the GluA1 subunit as a therapeutic target for epilepsy.

Among the major Annexin family members (ANXA1‐12), ANXA2 is a calcium‐dependent phospholipid‐binding protein.^15^ ANXA2 is expressed in the cortical and hippocampal tissues, where it is involved in various cellular functions such as calcium signaling, cell growth, vesicle transportation, and cell division.^16^, ^17^ Earlier studies have reported that dendrites and axons are enriched in ANXA2, where it participates in the regulation of axon growth and excitatory neuronal activity.^18^, ^19^ However, it is unknown whether ANXA2 can modulate the pathophysiological process of seizures by regulating excitatory neural activity.

In this study, the protein expression of ANXA2 was detected in intractable TLE patients, in epilepsy mouse models, and in vivo hippocampal neurons' model. Moreover, the effects of ANXA2 on epilepsy were examined through behavioral and electrophysiological analyses of KA‐induced mouse. Lastly, the potential molecular mechanism by which ANXA2 tunes epileptic susceptibility was discussed further.

## 2 MATERIALS AND METHODS

### 2.1 Human cerebral tissue

A diagnosis of refractory TLE was made in patients based on the criteria established by the International League Against Epilepsy. Regarding the epilepsy group, and 15 temporal samples of neocortex were acquired from patients with refractory TLE. Twelve patients with severe traumatic brain injury were sampled as the control group for brain tissue. All cerebral tissues were collected from the Second Hospital Affiliated to AMU and the First Affiliated Hospital of Chongqing Medical University. All patients from the control group had no history of epilepsy, seizures, antiepileptic treatment, or other central nervous system disorders. The collection of samples has been previously described.^20^, ^21^ The clinical characteristics of patients, such as gender, age, course, and AEDs before surgery, are presented in Table 1.

**TABLE 1:** Clinical characteristics of TLE and control patients.

Subject (No.)	Gender (M/F)	Age (Y)	Course (Y)	AEDs before the surgery	Resection tissue	Pathological diagnosis
P1	F	25	13	LTG, TPM, CBZ	RTN	G
P2	F	28	6	OXC, VPA, LTG	LTN	G, NL
P3	M	22	8	CBZ, VPA, PB	LTN	G, NL, ND
P4	M	23	7	VPA, LEV, PHT	LTN	G, NL
P5	F	28	11	VPA, CBZ, PHT	RTN	G, NL
P6	F	30	8	CBZ, PB, LTG, LEV	RTN	G, NL
P7	M	25	9	LTG, TPM, CBZ	RTN	G, NL
P8	F	17	5	VPA, CBZ, TPM	RTN	G, NL, ND
P9	M	27	9	OXC, VPA, PHT	LTN	G, NL, ND
P10	F	19	4	OXC, VPA, GBP	RTN	G, NL
P11	M	17	3	CBZ, VPA, PHT	LTN	G, NL
P12	F	23	5	CBZ, PHT, LTG	LTN	G, NL
P13	F	22	11	OXC, VPA, TPM	RTN	G, NL, ND
P14	M	23	6	CBZ, PHT, TPM	RTN	G, NL
P15	M	26	7	OXC, VPA, PHT	RTN	G, NL
C1	F	23	0	Brain trauma	RTN	N
C2	M	28	0	Brain trauma	LTN	N
C3	F	28	0	Brain trauma	RTN	N
C4	M	29	0	Brain trauma	LTN	N
C5	F	31	0	Brain trauma	LTN	N
C6	M	37	0	Brain trauma	RTN	N
C7	M	17	0	Brain trauma	LTN	N
C8	F	31	0	Brain trauma	RTN	N
C9	F	34	0	Brain trauma	LTN	N
C10	M	27	0	Brain trauma	RTN	N
C11	F	22	0	Brain trauma	RTN	N
C12	M	16	0	Brain trauma	LTN	N

Abbreviations: AEDs, antiepileptic drugs; C, control; CBZ, carbamazepine; CZP, clonazepam; F, female; G, Gliosis; L, left; LTG, lamotrigine; LVE, levetiracetam; M, male; N, normal; ND, neuron degeneration; NL, neuron loss; OXC, oxcarbazepine; P, patients; PB, phenobarbital; PHT, phenytoin; R, right; TN, temporal neocortex; TPM, topiramate; VPA, valproate; Y, years.

### 2.2 Animals

All experimental animals used in our study were acquired from Chongqing Medical University Experimental Animal Centre. We selected C57BL/6 mice, all of which were adult males (8–10 weeks old, weighing 20–25 g). Mice were treated under standard laboratory conditions at 22 ± 1°C with 50%–60% humidity in a light and dark cycle, each for 12 h with plenty of water and food.

### 2.3 KA‐induced epilepsy model and behavioral recordings

The intra hippocampal delivery process has been described in previous studies.^22^ Briefly, pentobarbital sodium (50 mg/kg, Sigma‐Aldrich) was utilized to anesthetize the mice via intraperitoneal injection prior to KA administration. Then, 20 mM solution KA (50 nL; Sigma‐Aldrich) was injected into the right hippocampal CA1 region (Stereo coordinates: anterior–posterior, 2.0 mm; inboard–outboard, 1.5 mm; dorsal‐abdomen, 1.5 mm) with a 0.5‐μL syringe (Hamilton) for 3 min. Then, the syringe was placed for 5 min and slowly withdrawn to reduce refluxes. The control group was injected in the same way by the 50 nL 0.9% normal saline.

The spontaneous recurrent seizures (SRSs) in mice were continuously monitored for 30 days by a video recording system. KA‐induced seizures were classified by Racine's criteria (stages 1–5). All mice in epilepsy groups reached the SRS stage 4–5.^23^ Our main findings were the latency period, number of SLEs, and Racine stage of SRSs, which were simultaneously confirmed and estimated independently by two investigators. After behavioral recording, hippocampal LFP was monitored to prove the successful construction of the KA‐induced mouse epilepsy models. A hole was drilled into the skull in the right area of CA1. The electrode was then placed in the hole and connected to a signal connector, which was secured with an acrylic dental cement.^24^ MAP Data Acquisition System (Plexon) and Neuroexplorer software (Nex Technologies) were separately used to monitor and analyze LFP. Seizure‐like events (SLEs) were defined above 5 Hz, amplitude two times higher than baseline, and discharge clusters that lasted longer than 5 s.^25^

### 2.4 Lentiviral (LV) constructs and stereotaxic injection

Lentivirals were constructed by GK Biotechnology, and used with a titer of 5 × 10^8^ TU/mL. ANXA2 knockdown was accomplished with LV vectors (GTATGATGCTTCGGAACTAAA) as the LV‐shANXA2 group, while LV scramble vectors (TTCTCCGAACGTGTCACGT) as the Con‐shRNA group. The green fluorescent protein (GFP) was expressed in all LV constructs. After stereotactically injecting the LV (2.5 μL, 0.25 μL/min) into the bilateral CA1 hippocampal area with a 5‐μL syringe, the syringe was left in place for 5 min prior to its slow withdrawal to prevent reflux. After continuous SRS observation for 30 days, LFP recordings were performed on mice in the control, con‐shRNA, and LV‐shANXA2 groups 7 weeks after injection of the LV vectors.^26^

### 2.5 Primary neuron culture and LV transfection

We prepared hippocampal neurons from C57BL/6 mice at postnatal days 0–1. The hippocampus was extracted, digested with trypsin for 10 min, mechanically triturated, centrifuged (1000 *g*, 5 min), triturated again, and stirred well. The dilution and plating of cell suspensions were performed in poly‐Lysine‐coated dishes, nurtured in DMEM (Gibco) consisting of 10% fetal bovine serum (Scitcher) and grown in incubator at 37°C for 4–6 h followed by replacement of medium with neurobasal medium, 1% glutamine, 1% antibiotic and 2% B27 (Gibco). Then, half of the medium capacity was changed every 3 days.^27^ After 10 DIV, extracellular free solution Mg^2+^ (145 mM NaCl, 10 mM glucose and HEPES, 2.5 mM KCl, 2 mM CaCl_2_, 0.002 mM glycine; osmotic pressure adjusted to 320 mOsm; pH 7.3) was used for the construction of the in vitro epilepsy model. The control group included 1 mM MgCl_2_ in additional extracellular solution. A fresh neurobasal medium replaced the extracellular solution for 24 h, and the neurons were collected for the next experiments. To knockdown ANXA2 expression in vitro at 3 DIV, the neurons were transfected with LV‐shANXA2, while the neurons in the negative control group were transfected with the control shRNA (Con‐shRNA).

### 2.6 Immunofluorescent staining

The immunofluorescence procedure has been previously described.^21^ Both mouse and human cerebral tissues were immobilized by 4% paraformaldehyde for 24 h, dehydrated 24 h by 10% and 30% sucrose solutions, respectively, and then cut into 16‐μm frozen sections. The frozen sections were retrieved in sodium citrate buffer and heated for 10 min at high temperature followed by 10 min at low temperature in the microwave and then left to naturally cool down. The sections were rinsed three times with PBS, incubated for 20 min at 37°C in Triton X‐100solution (0.4%; Beyotime), washed with PBS again, and incubated at 37°C with 5% goat serum (Boster) for 1 h. Next, the slices were incubated overnight in the following primary antibodies at 4°C: Mouse Annexin A2 monoclonal antibody (1:200, Proteintech, 66035‐1‐Ig); Rabbit Annexin A2 polyclonal antibody (1:200; Proteintech, 11256‐1‐AP); Rabbit NeuN polyclonal antibody (1:200, Zen Bio 381075); Rabbit GFAP polyclonal antibody (1:50, Proteintech, 16825‐1‐AP); Rabbit PSD95 polyclonal antibody (1:50, Proteintech, 20665‐1‐AP); Rabbit Synaptoporin polyclonal antibody (1:50, Proteintech, 14143‐1‐AP); Mouse GluA1 monoclonal antibody (1:200, Proteintech, 67642‐1‐Ig); Rabbit PKA polyclonal antibody (1:50, Proteintech, 12232‐1‐AP); Mouse PKC monoclonal antibody (1:50, Santa Cruz, sc‐17769); Rabbit CaMKII monoclonal antibody (1:200, Abcam, ab134041); and Mouse PP1 monoclonal antibody (1:50, Santa Cruz, sc‐7482).

After that, the slices were exposed to the following secondary antibodies at 37°C for 1 h: Cy3‐labeled Goat Anti‐Rabbit IgG (1:200, Beyotime, A0516); fluorescein isothiocyanate (FITC)‐labeled goat anti‐mouse IgG (1:200, Beyotime, A0568); Cy3‐labeled Goat Anti‐Mouse IgG (1:200, Beyotime, A0507); and Alexa Fluor 488‐labeled Goat Anti‐Rabbit IgG (1:200, Beyotime, A0423). At last, the slides were sealed using a fluorescence quencher containing DAPI.

For spine density analysis,^28^, ^29^ the cultured primary neurons were plated on coverslips. After 16–18 DIV, they were fixed for 30 min with 4% polyformaldehyde at 37°C, washed with PBS three times, permeabilized for 30 min by 0.1% Triton X‐100, rinsed again and then incubated at 37°C with 4% goat serum for 1 h. Subsequently, the control and ANXA2 knockdown groups did not require additional fluorescent staining because the lentiviral vector itself carried GFP fluorescent tags, while the normal control group was incubated overnight at 4°C with rabbit anti‐GFP antibodies (1:500, Abcam). Fluorescence images were obtained using a laser scanning confocal microscope (Andor or Nikon), and immunofluorescence analysis was performed using ImageJ software.

### 2.7 Western blot analysis and co‐immunoprecipitation

The Western blot methods used in this study were previously described.^20^ Membrane proteins of the mouse hippocampal tissue were collected using the Plasma Membrane Protein Isolation and Cell Isolation Kit (Invent). The following primary mouse monoclonal antibodies were employed: p‐CaMKII (1:1000, Santa Cruz, sc‐5306), PKC (1:1000, Santa Cruz, sc‐17769), PP1 (1:1000, Santa Cruz, sc‐7482); Na^+^‐K^+^‐ATPase (1:1000, Santa Cruz, sc‐48345), GluA1(1:10,000, Proteintech, 66035‐1‐Ig); rabbit monoclonal antibodies: AMPA subtype (phospho S845, 1:1000, Abcam, ab76321; phospho S831, 1:1000, Abcam, ab109464),PKA (phospho T197, 1:1000, Abcam, ab75991), CaMKII (1:1000, Abcam, ab134041), PKC (phospho T514, 1:1000, Abcam, ab109539); rabbit polyclonal antibodies: Annexin A2 (1:3000, Proteintech, 11256‐1‐AP); PKA (1:1000, Proteintech, 12232‐1‐AP); Beta Tubulin (1:5000, Proteintech, 10094‐1‐AP). The membrane was imaged with a Fusion imaging system (Vilber Lourmat).

The modified RIPA solution (Beyotime) was used to lyse hippocampal tissue from epileptic mice for co‐immunoprecipitation.^22^, ^30^ Based on the MedChem Express Protein A/G magnetic Beads manual, the rabbit IgG monoclonal antibody (Proteintech), the rabbit Annexin A2 polyclonal antibody (Proteintech), the mouse GluA1 monoclonal Antibody (Proteintech), the rabbit PKA polyclonal antibody (Proteintech), the rabbit CaMKII monoclonal antibody (Abcam), the mouse PKC monoclonal antibody (Proteintech), and the mouse PP1 polyclonal antibody (Santa Cruz) were incubated with 40 μL protein A/G magnetic beads at 4°C for 2 h. The hippocampal tissue supernatant was collected by centrifugation and then incubated with the antibody–bead complex at 4°C overnight. Subsequently, they were rinsed with phosphate buffer solution and analyzed by Western blotting or mass spectrometry. Samples of the target proteins and IgG electrophoresis gels were sent to Applied Shanghai Protein Technology after being silver‐dyed SDS‐PAGE for LC–MS/MS analysis.^31^

### 2.8 Electrophysiological recordings

The LV‐treated mice brains were quickly removed after anesthetization, and then sliced into 350‐μm‐thick coronal slices using a vibrating blade slicer (VT1200S; Leica). The sections were immersed in a recording tank, containing artificial cerebrospinal fluid (ACSF) with 125 mM NaCl, 2.5 mM KCl, 2 mM CaCl_2_, 1.25 mM NaH_2_PO_4_, 25 mM NaHCO_3_, 10 mM glucose, and 1 mM MgCl_2_. The pyramidal neurons in the CA1 region were observed with an inverted microscope and recorded with whole‐cell patch clamps.^24^ The miniature excitatory postsynaptic current (mEPSCs) was recorded with an electrode in a solution containing 130 mM CsMeSO_4_, 12 mM Na‐phosphocreatine, 10 mM HEPES and CsCl, 5 mM Mg‐ATP and NMG, 4 mM NaCl, 1 mM MgCl_2_·6H_2_O and EGTA, 0.5 mM Na3‐GTP, 285 mM mOsm, pH 7.2.When the miniature inhibitory postsynaptic current (mIPSC) was recorded, the electrode internal solution was composed of 110 mM CsCl, 30 mM NMG, 12 mM Na‐phosphocreatine, 10 mM HEPES, 5 mM Mg‐ATP, 1 mM MgCl_2_·6H_2_O, and EGTA, 0.5 mM Na3‐GTP, 290 mOsm, pH 7.2.^32^ When the action potential (AP) was recorded, the electrode internal solution comprised 60 mM K_2_SO_4_ and NMG, 40 mM HEPES,12 mM Na‐phosphocreatine, 2 mM Na2‐ATP, 0.2 mM Na3‐GTP, 4 mM MgCl_2_·6H_2_O, 0.5 mM BAPTA, 280 mOsm.^28^ The electrophysiological data were collected using a Multiclamp 700B amplifier (Axon) and Digidata 1550B interface (Axon) and analyzed by pCLAMP 9.2 software (Molecular Devices).

### 2.9 Statistical analysis

All data were tested for normality using the Shapiro–Wilk test, and the variance homogeneity was used for the Levene's test. If the data were normal and uniform, they were expressed as mean standard error (mean ± SD). Unpaired Student's two‐tailed *t*‐test and Fisher's exact test or one‐way ANOVA was used for statistical evaluation (unpaired Student's two‐tailed *t*‐test was used for comparing between two groups, one‐way analysis of variance ANOVA followed by the Bonferroni post hoc test was used for comparing between multiple groups (more than two groups)). If the data were non‐normally distributed or inhomogeneous were presented as median and interquartile range (IQR) and statistically analyzed using the Kruskal–Wallis nonparametric test. *p* < 0.05 was deemed statistically significant. All statistical tests were conducted by the SPSS 22.0 software.

## 3 RESULTS

### 3.1 ANXA2 expression in TLE patients

**FIGURE 1:**
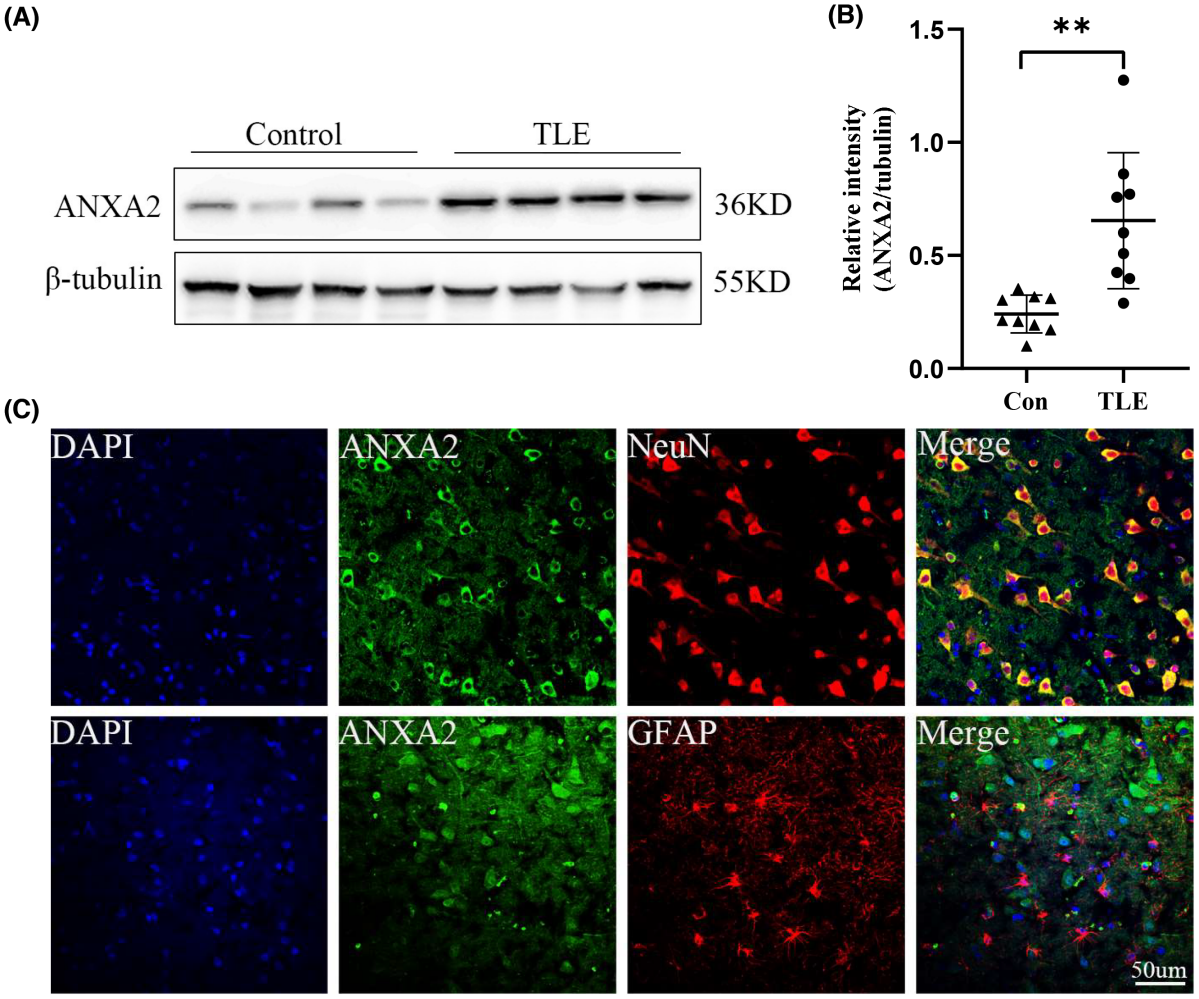
ANXA2 expression in TLE patients and control individuals. (A, B) Western blot showing that ANXA2 protein level is elevated in the neocortex of TLE patients (*n* = 9) compared with that of the control group (*n* = 9) (**p = 0.0011, unpaired *t*‐test). (C) Dual immunofluorescence image showing that ANXA2 (green) colocalizes with the neuronal marker NeuN (red) but not with the astrocyte marker GFAP (red) in TLE patients (scale bar = 50 μm). **p < 0.01.

In this study, there were seven male and eight female patients in the TLE group, and their average age was 23.67 ± 3.92 (range 17–30 years). Meanwhile, six male and six female patients were included in the control group, and the average age was 26.92 ± 6.39 (range 16–37 years). Sex and age did not statistically differ between the two groups (Table 1). First, we detected changes in ANXA2 protein expression levels in TLE patients and controls by Western blotting. The expression level of ANXA2 was higher in the TLE group than in the control group (Figure 1A,B). Immunofluorescence staining in the TLE tissue slices showed that ANXA2 was predominantly localized in neurons. As shown in Figure 1C,D, ANXA2 co‐localizes with NeuN (a neuronal marker), but not with GFAP (an astrocyte marker).

### 3.2 ANXA2 expression in epileptic models

**FIGURE 2:**
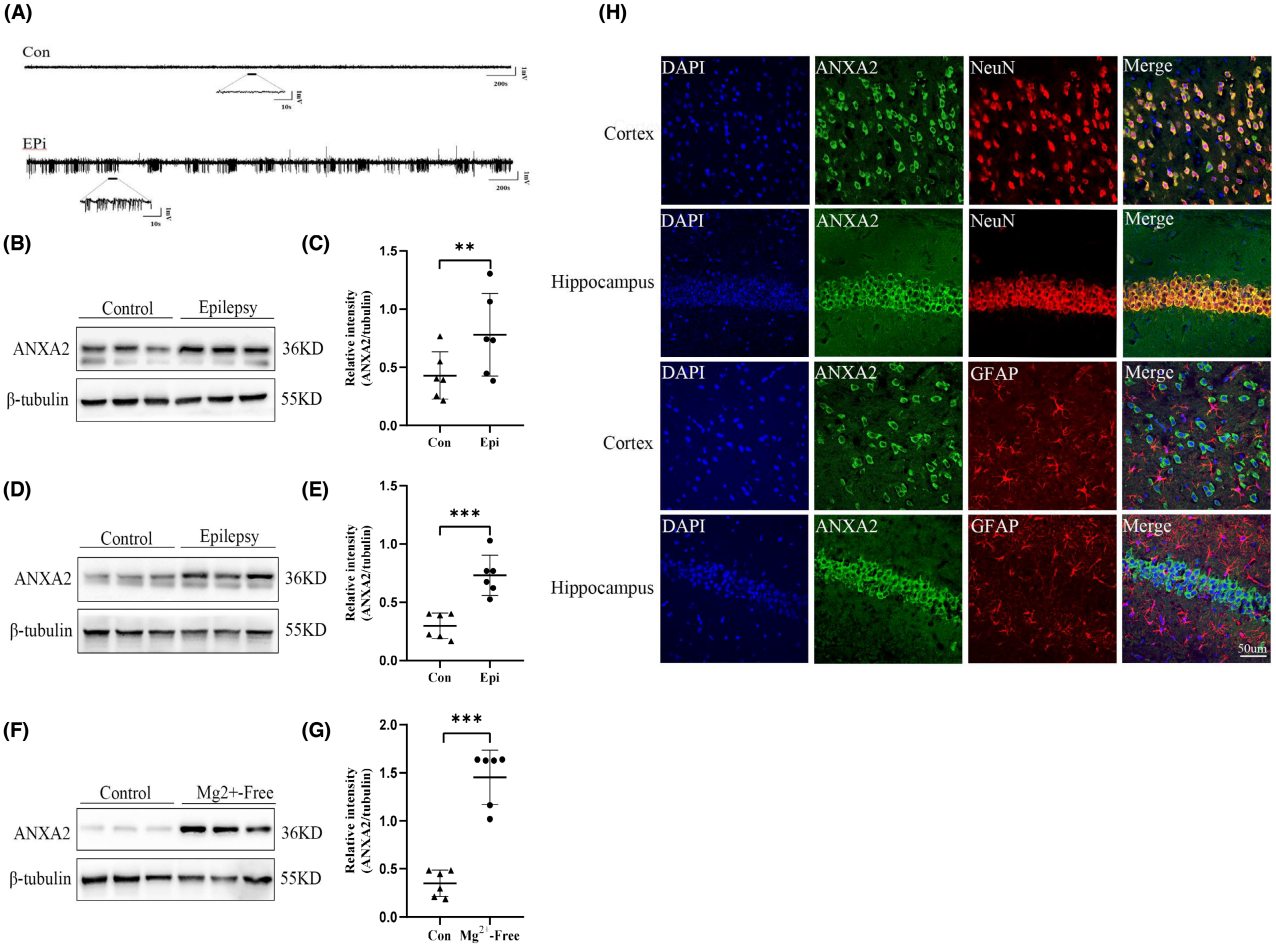
ANXA2 expression and localization in the temporal cortex and hippocampus of KA‐induced epilepsy mice. (A) Representative LFP images. Western blots showing that the epilepsy group (*n* = 6) has a significantly higher expression of ANXA2 in the temporal cortex (B, C) (**p = 0.0017, unpaired *t*‐test), hippocampus (D, E) (***p = 0.0004, unpaired *t*‐test) and Mg^2+^‐free model (F, G) (***p = 0.0002, unpaired *t*‐test) in comparison with the control group (*n* = 6). (H) Representative dual immunofluorescence imaging showing that ANXA2 (green) and NeuN (red) are co‐expressed, but ANXA2 is not co‐localized with GFAP (red) (scale bar = 50 μm). **p < 0.01, ***p < 0.001.

The construction of a mouse epilepsy model was confirmed via hippocampal LFP recordings (Figure 2A). In KA‐induced epilepsy mice, ANXA2 protein expression was significantly up‐regulated in the temporal cortex and hippocampus (Figure 2B–E). Similarly, ANXA2 protein expression was also markedly elevated in the Mg^2+^−free neurons in compared with the control neurons (Figure 2F,G). Consistent with the results of immunofluorescence analysis of TLE specimens, ANXA2 was coexpressed only with NeuN cells, but not with GFAP cells in the hippocampus and cerebral cortex of epileptic mouse model (Figure 2G).

### 3.3 Impact of ANXA2 knockdown on seizure activity in vivo

**FIGURE 3:**
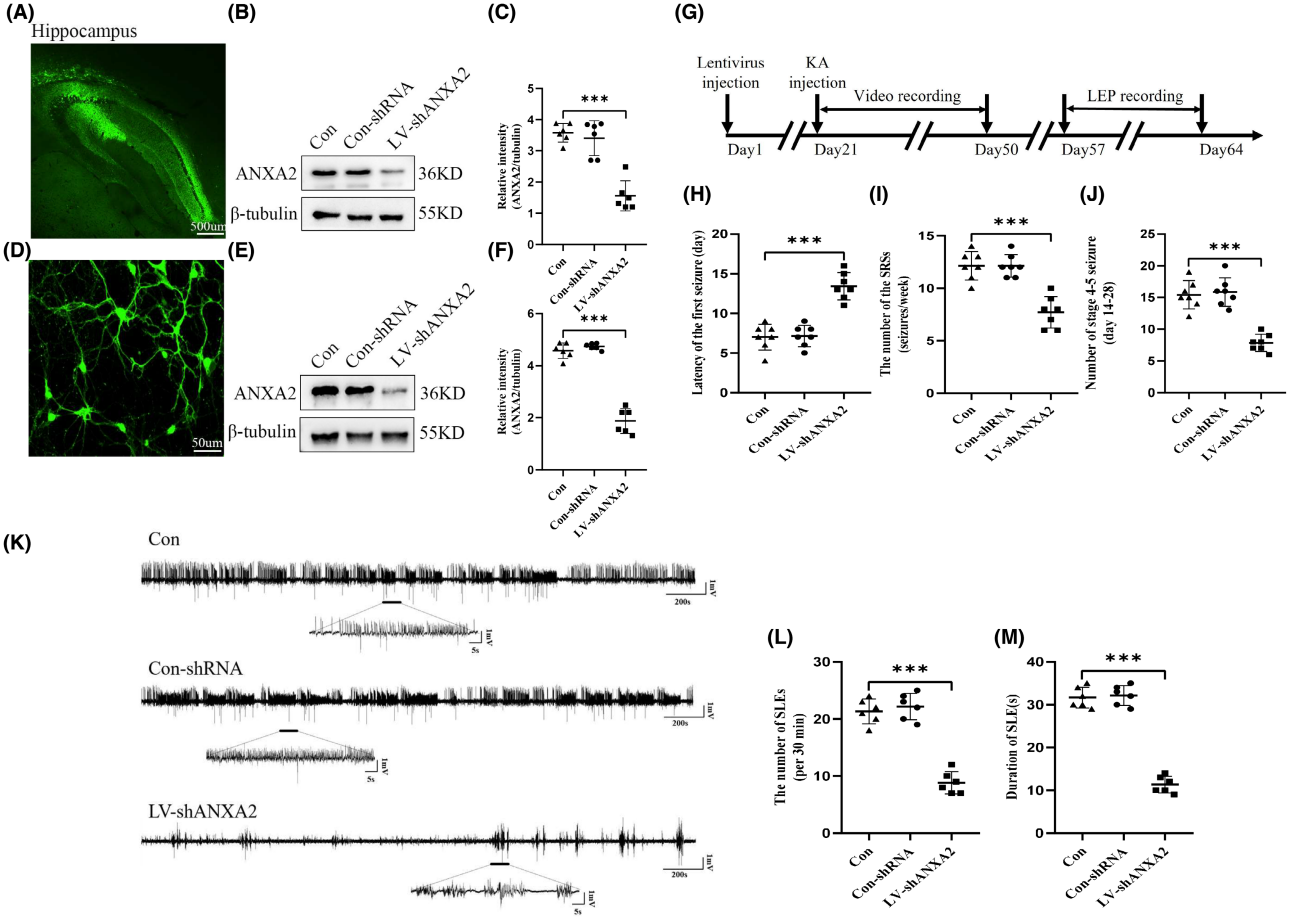
Impact of LV‐shANXA2 on the seizure susceptibility of KA‐induced mice. Image of LV expressing green fluorescent protein in the hippocampus (A) (scale bar = 500 μm) at week 3 and in the cultured neurons (D) (scale bar = 50 μm) at 14 DIV. Western blots showing the reduced ANXA2 expression in the LV‐shANXA2 group (*n* = 6) compared with that in the Con‐shRNA (*n* = 6) and control group (*n* = 6) in vivo (B, C) and in vitro (E, F). (G) Experimental roadmap. Statistical charts of the latency period (H), the SRSs number per week (I), and the stage 4–5 number of SRSs (J), showing that the LV‐shANXA2 group (*n* = 7) has a prolonged latency, a decreased SRS count, and a suppressed seizure severity compared with those of control group (*n* = 7). (K) Representative LFP images. Statistical analysis showing a decrease in the occurrence (L) and duration (M) of SLE in the LV‐shANXA2 group (*n* = 6) compared with that in the Con‐shRNA (*n* = 6) and control group (*n* = 6). ***p < 0.001, One‐way analysis of variance (ANOVA) performed first, followed by Tukey's test.

To determine whether the changes in ANXA2 levels can affect seizure activity, the LVs was administered 3 weeks before KA‐induced chronic seizures. We first observed the auto fluorescence of LVs GFP in the hippocampal region, confirming the successful infection of mice with the lentiviruses (Figure 3A). LVs were also used to successfully infect primary cultured hippocampal neurons 14 DIV (Figure 3D). Subsequently, Western blotting was conducted to determine the effect of virus transfection. As expected, ANXA2 protein expression in the LV‐shANXA2 group was remarkably lower than that in the Con‐shRNA and control groups in vivo (Figure 3B,C) and in vitro (Figure 3E,F). The results of the appeal showed that LV‐shANXA2 virus was successfully transfected in mice and cell models of epilepsy. Four weeks after KA injection, the behaviors of mice were continuously monitored to evaluate their seizure susceptibility (Figure 3G). Behavioral analysis demonstrated that compared to the Con‐shRNA and control groups, the LV‐shANXA2 group has a prolonged first seizure latency (Figure 3H), a reduced number of SRSs (Figure 3I), and a number of grade 4–5 SRSs (Figure 3J). The LFP recordings in the mouse hippocampus (Figure 3K–M) showed that the LV‐shANXA2 group has lesser and shorter SLEs compared with the Con‐shRNA and control groups. Meanwhile, seizure susceptibility and SLEs were not different between the Con‐shRNA and control groups. This indicates that seizure activity is suppressed by ANXA2 knockdown in the epileptic mice.

### 3.4 Inhibition of ANXA2 results in decreased glutamatergic synaptic transmission

**FIGURE 4:**
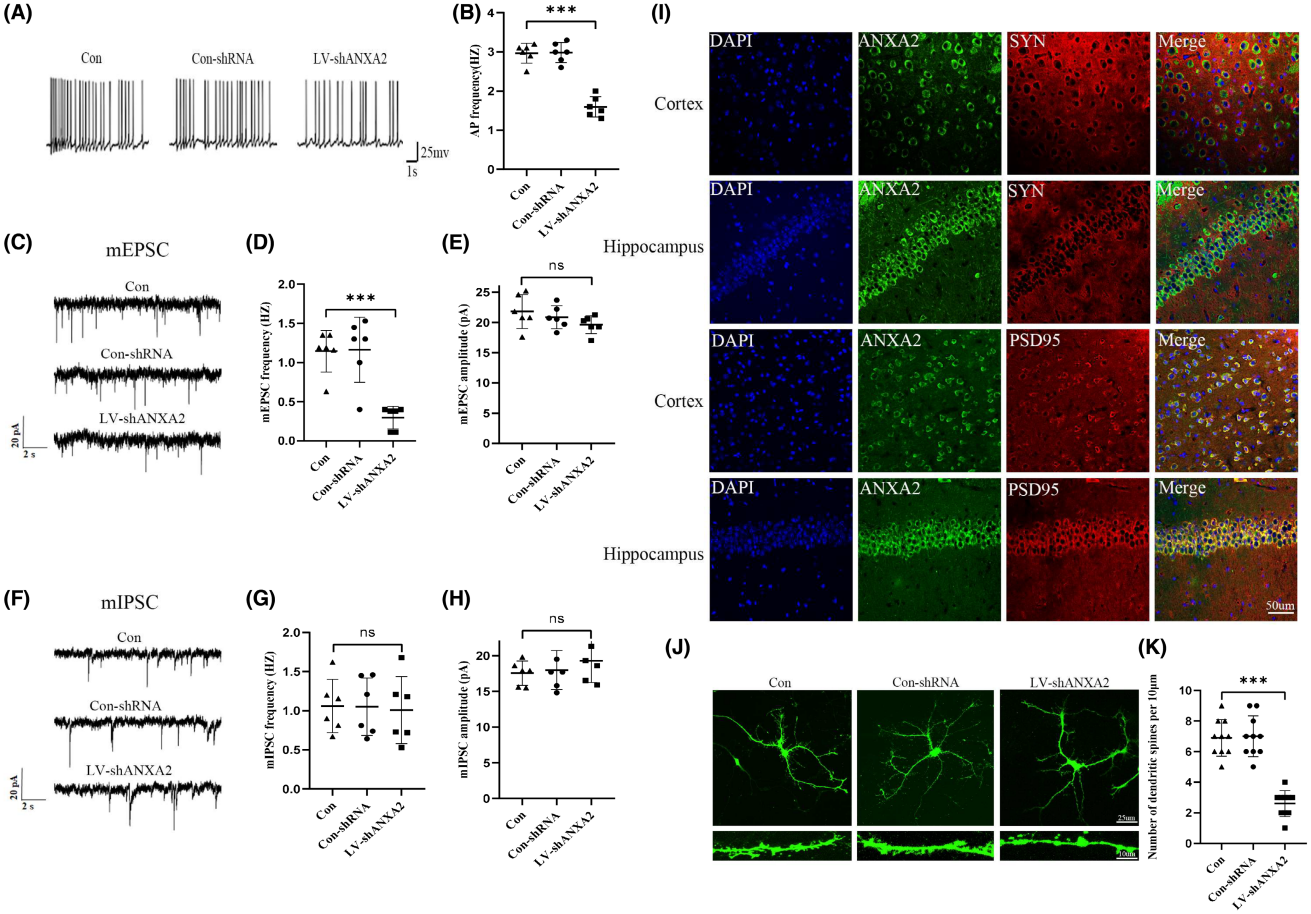
Impact of LV‐ANXA2 on KA‐induced excitatory synaptic transmission in mice. (A) Representative AP graph. (B) Spontaneous AP incidence was lower in the LV‐shANXA2 group (*n* = 6) than Con‐shRNA (*n* = 6) and control group (*n* = 6). (C) Representative mEPSC images. ANXA2 knockdown significantly decreased mEPSC frequency (D) but did not influence mEPSC amplitude (E), compared with the other groups (*n* = 6 each group). (F) Representative mIPSC images. The frequency (G) and amplitude (H) of mIPSCs in KA‐kindled mice were not different in all groups (*n* = 6 each group). (I) Dual immunofluorescence showing that ANXA2 (green) and PSD95 (red) expressions are merged, while no merging was observed with SYP (red) (scale bar = 50 μm). (J) Representative images of the dendrites in vitro. (K) The ANXA2 knockdown group had fewer dendritic spines than the other two groups (*n* = 10 per group). The top scale is 25 μm, and the bottom scale is 10 μm. ***p < 0.001, N.S., not significant, *p* > 0.05, ANOVA followed by Tukey's test.

First, the APs of pyramidal neurons in CA1 region induced by Mg^2+^ free ACSF were detected by whole‐cell patch‐clamp. The results showed that the AP frequency was reduced in LV‐shANXA2 mice compared with Con‐shRNA and untreated control mice (Figure 4A,B). Next, we explored whether the downregulation of ANXA2 affects postsynaptic transmission. Therefore, we recorded the mEPSCs and mIPSCs in the CA1 region pyramidal neurons of mouse hippocampus. Recordings of CA1 pyramidal neurons from LV‐shANXA2 mice showed a significantly lower mEPSCs frequency and no change in amplitudes compared with Con‐shRNA and untreated control mice (Figure 4C–E). Furthermore, mIPSCs had no significant changes in frequency and amplitude (Figure 4F–H). To further investigate the localization of ANXA2 in excitatory synapses, immunofluorescence staining revealed that ANXA2 was localized to PSD‐95 (apostsynaptic excitatory marker), but not to synaptophysin (SYP, a presynaptic marker) (Figure 4I). Taken together, these data suggest that ANXA2 primarily mediates glutamatergic excitatory postsynaptic function in epileptic mice.

Dendritic spine density has been reported to be associated with excitatory synapses.^33^ Therefore, we detected whether ANXA2 knockdown leads to dendritic spine changes in cultured hippocampal cells by immunofluorescence staining. Consistent with the results of excitability reduction in the patch‐clamp LV‐shANXA2 group, the dendritic spine density of the LV‐shANXA2‐intervened neurons was markedly lower than that of the control neurons (Figure 4J,K).

### 3.5 ANXA2 inhibition downregulates surface expression of GluA1 by regulating serine 831 and 845 phosphorylation

**FIGURE 5:**
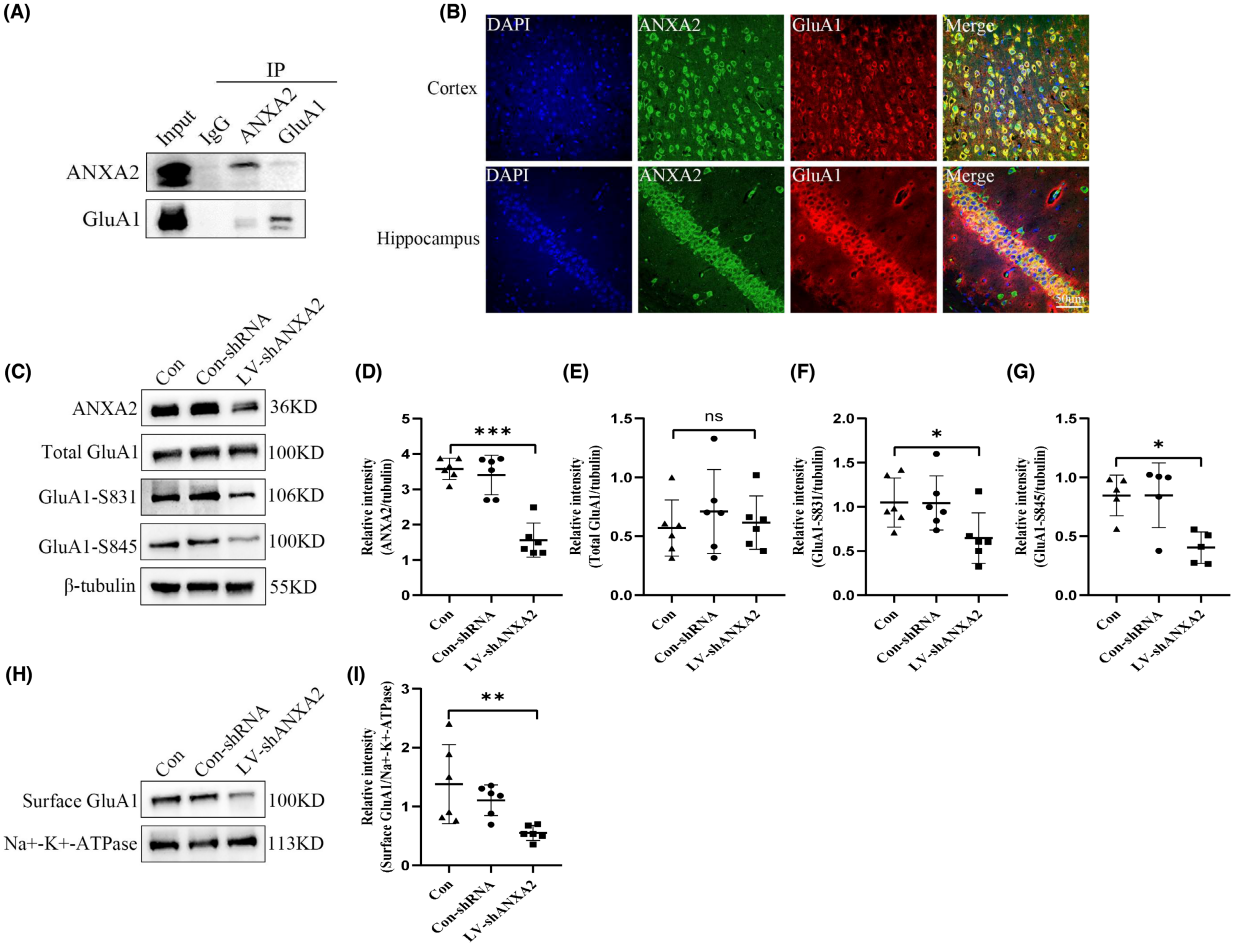
Influence of ANXA2 on GluA1 cell surface expression. (A) Results of ANXA2 and GluA1 COIP assays in the hippocampus. (B) Dual immunofluorescence imaging of ANXA2 and GluA1 in epilepsy mice. ANXA2 (green) and GluA1 (red) are merged (scale bar = 50 μm). (C–I) Western blots images and statistical analysis of the expression of total GluA1, cell surface GluA1, GluA1‐s831, and GluA1‐s845 (*n* = 6 per group). *p < 0.05, **p < 0.01, ***p < 0.001, N.S., not significant, *p* > 0.05. ANOVA followed by Tukey's test.

To identify ANXA2 interacting proteins in hippocampal tissue from KA‐induced epilepsy mice, mass spectrometry coupled co‐immunoprecipitation mass spectrometry (COIP‐MS) was conducted. Consistent with the COIP‐MS results (Table S1), we found that the GluA1 subunit of AMPAR interacts with ANXA2. This interaction was confirmed by COIP, indicating that GluA1 is a downstream target of ANXA2 in our epilepsy model (Figure 5A). Meanwhile, immunofluorescence staining showed that ANXA2 colocalizes with GluA1 (Figure 5B). Next, the impact of ANXA2 downregulation on GluA1expression in the hippocampus was determined by Western blotting. Surprisingly, the downregulation of ANXA2 did not reduce total GluA1 expression (Figure 5C–E), but decreased its surface expression (Figure 5H,I). GluA1 is known to be one of the important AMPAR subunits involved in regulating excitatory synaptic transmission in epilepsy. Therefore, we speculate that ANXA2 may mediate excitatory postsynaptic function by regulating GluA1. It has been demonstrated that GluA1 is mainly distributed on cell membranes by phosphorylating on serine 845 and 831.^34^, ^35^ Meanwhile, S831‐ and S845‐mediated GluA1 phosphorylation is associated with an increase in EPSCs mediated by AMPAR.^36^ Therefore, we further investigated the expression levels of GluA1‐S831 and GluA1‐S845 in mice with epilepsy after LV‐ANXA2 intervention and found that their expression levels were significantly decreased (Figure 5C,F–D). In summary, we hypothesize that ANXA2 regulates the surface expression of GluA1 via affecting the phosphorylation levels of GluA1‐S845 and GluA1‐S831, thereby mediating excitatory synaptic transmission.

### 3.6 ANXA2 modulates S831 phosphorylation level by influencing PKC activity and S845 phosphorylation level through affecting PKA activity

**FIGURE 6:**
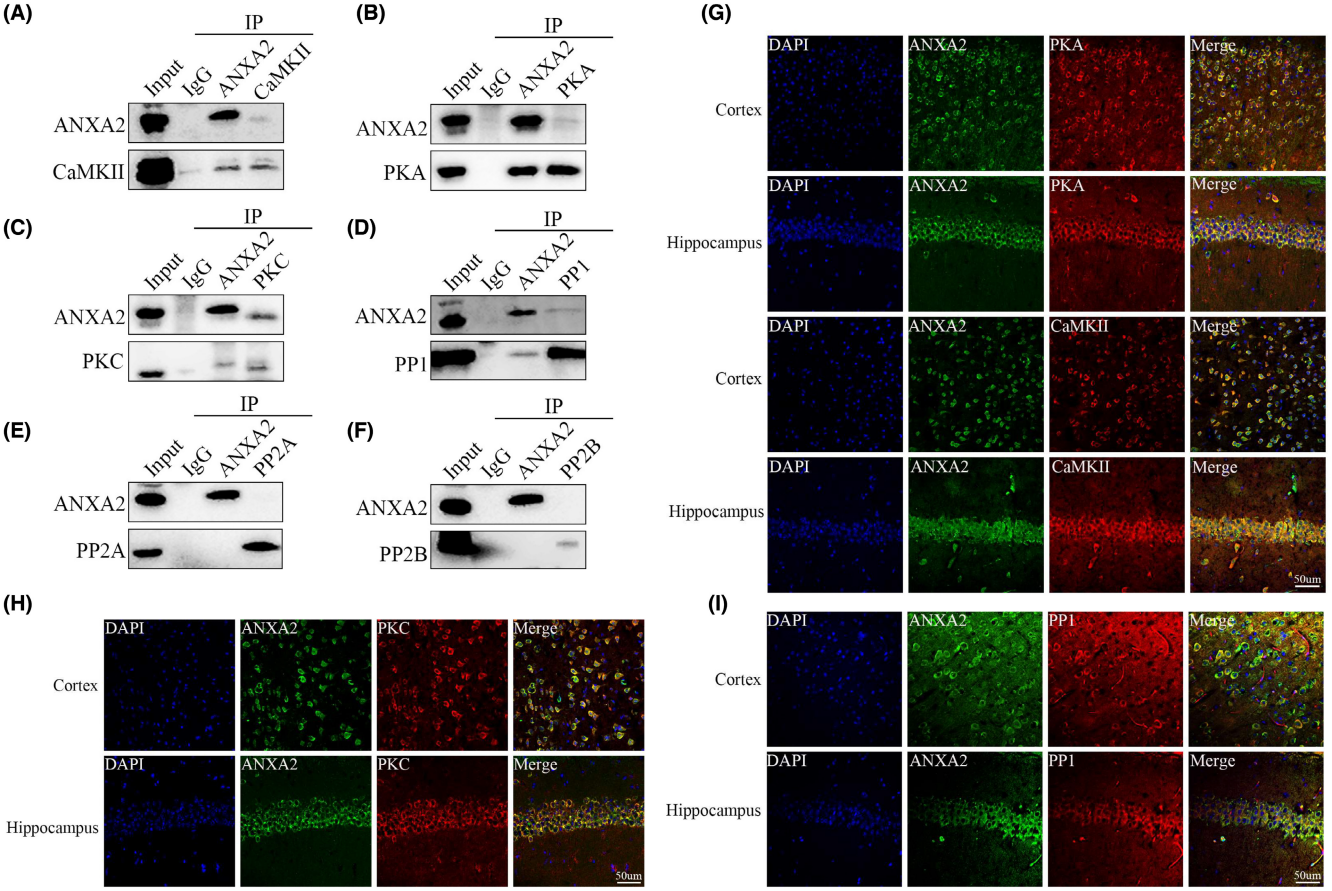
Effects of ANXA2 on GluA1 phosphorylation. (A–F) COIP maps showing that ANXA2 interacts with CaMKII, PKA, PKC, and PP1 in the epilepsy mouse model, but not with PP2A and PP2B (*n* = 5 per group). (G–I) Dual immunofluorescence imaging of ANXA2 and PKA, PKC, CaMKII, and PP1. ANXA2 (green) and PKA, CaMKII, PKC, and PP1 (red) co‐localized (merged) (scale bar = 50 μm).

**FIGURE 7:**
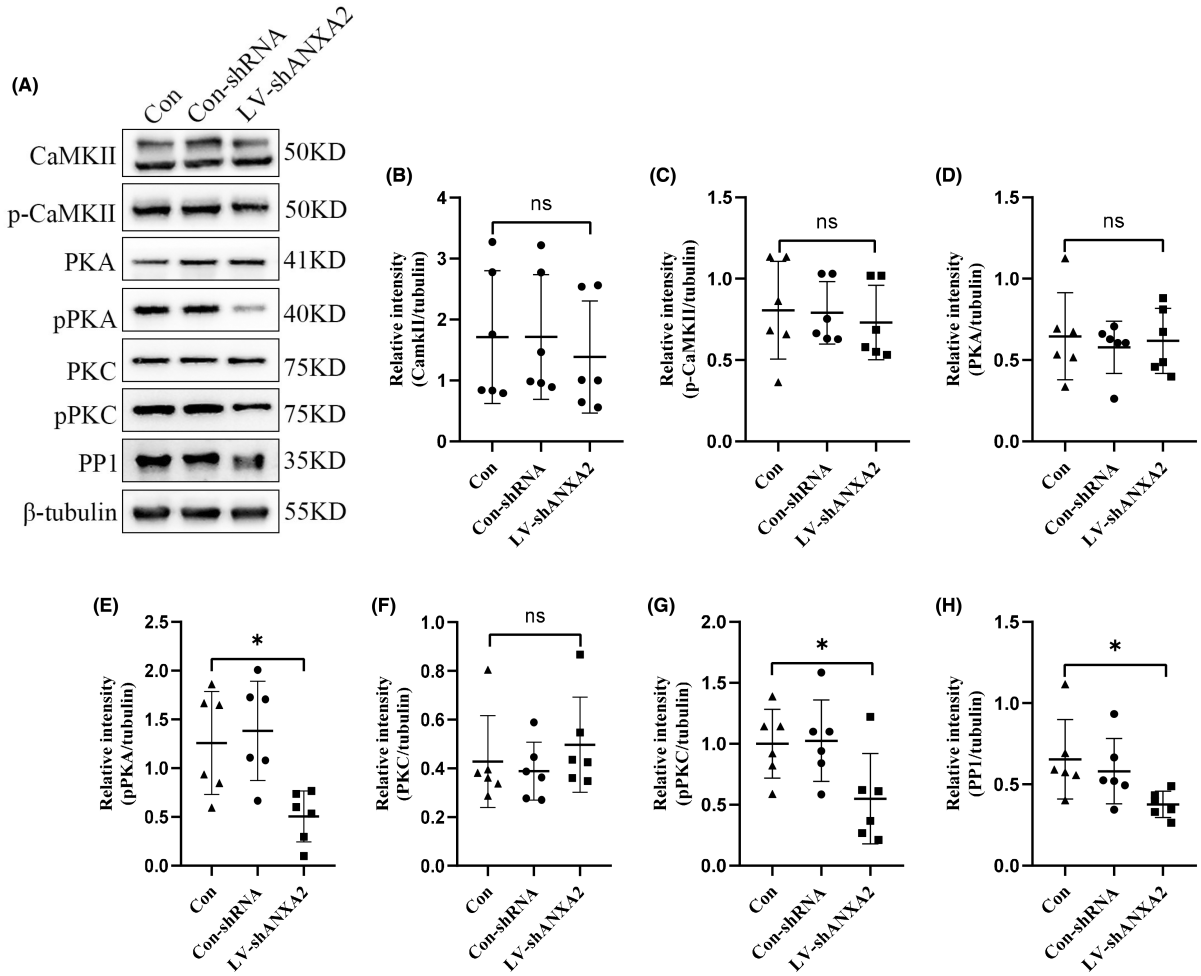
Effects of ANXA2 on the phosphorylation of GluA1 at serine 831 and serine 845. (A–H) Western blot images and statistical analysis of the expression of CaMKII, pCaMKII, PKA, pPKA, PKC, pPKC, and PP1 (*n* = 6 each group). *p < 0.05, N.S., not significant, *p* > 0.05, ANOVA followed by Tukey's test.

It has been reported that the phosphorylation of GluA1 on S831 was determined by PKC, and the phosphorylation of GluA1 on S845 was controlled by CAMKII and PKA. However, GluA1 dephosphorylation was mediated via PP1, PP2A, and PP2B.^37^ The combination of COIP and COIP‐MS results revealed that ANXA2 could interact with PKA, PKC, CAMKII and PP1, but not with PP2A and PP2B in the hippocampus of mice (Figure 6A–F). Immunofluorescence staining showed that ANXA2 colocalizes with PKA, CAMKII, PKC and PP1 (Figure 6G–I). The down‐regulation of ANXA2 expression in the hippocampi reduced the levels of phospho‐PKA (pPKA), phospho‐PKC (pPKC) and PP1, but did not change the expression of phospho‐CaMKII (pCaMKII) (Figure 7A–H). Based on the data that the expression levels of S845 and S831 were reduced after ANXA2 knockdown, it was found that PKA‐targeted phosphorylation of GluA1 S845 and PKC‐targeted phosphorylation of GluA1 S831 were more effective than PP1 dephosphorylation in epilepsy.

## 4 DISCUSSION

This is, to our knowledge, the first study that describes the impact of ANXA2 on epilepsy. It was found that ANXA2 could regulate excitatory synaptic activity mediated by the GluA1 subunit of AMPAR, thus exacerbating seizure activity. First, elevated ANXA2 protein expression was detected in TLE patients and epilepsy models in vivo and in vitro. Subsequently, ANXA2 down‐regulation was found to inhibit epileptic activity and reduce abnormal discharge in behavioral analysis compared with control mice. Further studies showed that down‐regulation of ANXA2 significantly inhibited epileptic activity, in part, by down‐regulating GluA1 cell‐surface protein levels via reduced phosphorylation of S831 and S845.

ANXA2, an important annex in family member, was reported to be highly expressed in patients with tuberous sclerosis with seizures. However, it has not been studied in epilepsy.^18^, ^38^ Given that synaptic dysfunction is closely associated with epilepsy and that previous reports indicate that ANXA2 plays a major role in the development of excitatory synaptic activity, we speculate that ANXA2 can participate in epileptogenesis by regulating synaptic transmission. The changes in its postsynaptic potential were detected by patch clamp to further clarify whether ANXA2 participates in seizures across excitatory or suppressive synapses. Our subsequent electrophysiological studies revealed that tissue slices from LV‐shANXA2 mice have a significantly reduced mEPSC frequency. However, the mEPSC amplitude, mIPSC amplitude, and frequency did not significantly change. This suggests that ANXA2 may affect epileptogenesis by controlling the function of excitatory postsynaptic glutamate transmission rather than inhibiting the postsynaptic GABA transmission. Moreover, we found that Aps was significantly down‐regulated in the ANXA2 knockdown group compared to those in control groups. The excitability of axons can be reflected in the number of dendritic spines, and the reduced number of dendritic spines may attenuate the excitatory activity in the brain. Here, we demonstrated that ANXA2 knockdown decreased the number of dendritic spines, leading to a lower level of excitatory synaptic transmission. Meanwhile, our animal model studies showed a significant reduction in seizures and abnormal discharges in the LV‐shANXA2 animals. In summary, we can conclude that ANXA2 mainly mediates excitatory synaptic transmission activity in epilepsy and inhibiting ANXA2 activity has an antiepileptic effect.

AMPAR surface proteins, including GluA1, are dynamically changing; their distribution is tightly regulated, and AMPAR modification is largely dependent on serine phosphorylation of GluA1.^39^ Using an immunoprecipitation assay, we found that ANXA2interacts with GluA1 in epileptic hippocampal tissue. Furthermore, we observed that there was no alteration in total GluA1 protein, but found its cell membrane expression was significantly reduced in the hippocampal tissues of epileptic mice with ANXA2 knockdown. Previous studies have indicated that GluA1 accumulation on cell surfaces is largely dependent on the phosphorylation levels of GluA1 on S831 and S845.^39^ Interestingly we found that the phosphorylation levels of S831 and S845 were largely down‐regulated in KA‐induced hippocampal tissue with ANXA2 knockdown. Furthermore, ANXA2 knockdown reduced the phosphorylation activities of PKA and PKC, which are important promoters of GluA1 phosphorylation on serine 845 and serine 831. GluA1 is primarily phosphorylated at serine 845 and S831, and their phosphorylation levels largely affect GluA1 retention on the cell surface. Phosphorylation on serine 845 is promoted by PKA and CaMKII, and the phosphorylation on serine 831 is facilitated by PKC.^40^, ^41^ In the current study, it was found that ANXA2 interacted with PKA, PKC, and CaMKII by COIP. The inhibition of ANXA2 activity also down‐regulated the phosphorylation of PKA and PKC. However, the phosphorylation activity of CaMKII was not affected in epileptic mice. These results demonstrate that PKA and PKC can mediate ANXA2 regulation on S845 and S831. The dephosphorylation of GluA1 proteins through PP1, PP2A, and PP2B also influences the expression levels of S845 and S831. In the COIP experiments, ANXA2 interacted with PP1, but not with PP2A and PP2B. Interestingly, the expression level of PPI was also decreased after ANXA2 knockdown. Taken together, the phosphorylation mediated by PKA and PKC is more significant than the dephosphorylation mediated by PP1 during the process of GluA1 cell surface modification regulated by ANXA2.

Nevertheless, there are some limitations to this study that should be addressed. First, we did not test whether PKA and PKC regulate the cell membrane and total protein levels of GluA1 through in vivo or in vitro experiments. However, previous studies have shown that the phosphorylation of GluA1 is significantly inhibited after dephosphorylation of PKA or PKC. Second, this study mainly explored the role of ANXA2 in glutamate excitatory activity mediated by GluA1 and did not investigate other glutamate exciting receptors, which should be further studied. Third, although the KA‐induced epilepsy model is a recognized experimental model of temporal lobe epilepsy, it cannot fully reflect the complexity of clinical epilepsy.

## 5 CONCLUSIONS

By reporting a previously unrecognized and important role of ANXA2 in epilepsy, we first found that ANXA2 was highly expressed in the brain tissues of TLE patients and KA‐induced epileptic mice. Next, we observed that down‐regulation of ANXA2 expression could inhibit epileptic sensitivity and reduce the firing of abnormal neurons, suggesting that inhibition of ANXA2 activity could attenuate epileptic seizures. Further studies show that ANXA2 knockdown can reduce the cell membrane expression level of GluA1 and glutamate excitatory synaptic transmission activity, and ANXA2 regulates GluA1 activity mainly through PKC‐mediated phosphorylation of S831 and PKA‐mediated phosphorylation of S845. In conclusion, this research focuses on how ANXA2 mediates excitatory synaptic activity in epilepsy and also provides novel clues for alternative epilepsy therapies.

## AUTHOR CONTRIBUTIONS

LM, QW, and YC designed the experiments. LM, QW, JY, YW, PZ, QL, DT and ML carried out the experiments and analyzed the data. LM, QW, and JY drafted the manuscript. YC supervised the all tasks.

## FUNDING INFORMATION

This work was supported by the National Natural Science Foundation of China, Grant/Award Number: 82071458 and 81771390. This research was also supported by Chongqing Natural Science Foundation, Grant/Award Number: CSTB2022NSCQ‐MSX1203.

## CONFLICT OF INTEREST STATEMENT

The author claims no conflict of interest.

## CONSENT TO PARTICIPATION

Informed consent was signed by the patients or their guardians for the use of clinical data and brain tissues.

## CONSENT TO PUBLISH

No conflict of interest exits in submitting this manuscript.

## Supporting information


Table S1

## Data Availability

The data sets produced during the study period can be provided by the corresponding authors upon reasonable request.
